# Biological Aspects of Selected Myokines in Skeletal Muscle: Focus on Aging

**DOI:** 10.3390/ijms22168520

**Published:** 2021-08-07

**Authors:** Rosa Mancinelli, Franco Checcaglini, Francesco Coscia, Paola Gigliotti, Stefania Fulle, Giorgio Fanò-Illic

**Affiliations:** 1 Department of Neuroscience Imaging and Clinical Sciences, University “G. d’Annunzio” of Chieti-Pescara, 66100 Chieti, Italy; r.mancinelli@unich.it (R.M.); stefania.fulle@unich.it (S.F.); 2 IIM-Interuniversity Institute of Myology, University “G. d’Annunzio” of Chieti-Pescara, 66100 Chieti, Italy; 3 Free University of Alcatraz, Santa Cristina di Gubbio, 06100 Perugia, Italy; franco.ceccaglini@libero.it; 4 Department of Medicine, Laboratory of Sport Physiology, University of Perugia, 39038 San Candido-Innichen, Italy; francesco.coscia1@gmail.com (F.C.); pao.gigliotti@gmail.com (P.G.); 5 A&C M-C Foundation for Translational Myology, 35100 Padova, Italy

**Keywords:** sarcopenia, skeletal muscle secretome, physical activity, niche, satellite cells, oxidative stress

## Abstract

In the last decade, clear evidence has emerged that the cellular components of skeletal muscle are important sites for the release of proteins and peptides called “myokines”, suggesting that skeletal muscle plays the role of a secretory organ. After their secretion by muscles, these factors serve many biological functions, including the exertion of complex autocrine, paracrine and/or endocrine effects. In sum, myokines affect complex multi-organ processes, such as skeletal muscle trophism, metabolism, angiogenesis and immunological response to different physiological (physical activity, aging, etc.) or pathological states (cachexia, dysmetabolic conditions, chronic inflammation, etc.). The aim of this review is to describe in detail a number of myokines that are, to varying degrees, involved in skeletal muscle aging processes and belong to the group of proteins present in the functional environment surrounding the muscle cell known as the “Niche”. The particular myokines described are those that, acting both from within the cell and in an autocrine manner, have a defined relationship with the modulation of oxidative stress in muscle cells (mature or stem) involved in the regulatory (metabolic or regenerative) processes of muscle aging. Myostatin, IGF-1, NGF, S100 and irisin are examples of specific myokines that have peculiar features in their mechanisms of action. In particular, the potential role of one of the most recently characterized myokines—irisin, directly linked to an active lifestyle—in reducing if not reversing senescence-induced oxidative damage is discussed in terms of its possible application as an agent able to counteract the deleterious effects of muscle aging.

## 1. Background

In recent years, several studies have revealed a new role for skeletal muscle as a secretory organ since its contraction has emerged as an important activator of the release of proteins and peptides called “myokines”. These factors, with molecular weights of 5–20 kDa, are mainly secreted by skeletal muscle fibers and are capable of exerting many biological effects, either directly on the muscle that has secreted them (autocrine effect) or on tissues located nearby (paracrine effect) or, when transported by the blood, far from the site of production (endocrine effect), affecting complex multi-organ processes such as metabolism, angiogenesis and inflammation [1]. For these reasons, the current definition of myokines is “cytokines and other peptides that are produced, expressed and released by muscle fibers and exert either autocrine, paracrine, or endocrine effects” [2]. One of the best-known processes associated with autocrine/paracrine secretory activity concerns the mechanisms involved in controlling and restoring muscle mass in different conditions, both physiological, such as aging or microgravity, and pathological, such as diabetes, heart failure or cancer. The reduction in muscle size is known as hypotrophy and can result from a decrease in cell size due to either the loss of cellular content or an alteration in protein synthesis. This second event, in turn, may be due either to increased protein degradation through enhanced proteasomal and lysosomal activity via activation of FOXO-3 signaling or to reduced protein synthesis regulated mainly by the PI3K/AKT pathway [3]. The loss of muscle mass may also be due to a reduction in the number of fibers (hypoplasia) of a muscle, without a significant decrease in the trophic state of the remaining fibers [4]. However, the number of fibers constituting each muscle largely depends on the regenerative capacity of the tissue directly linked to the activity of the pool of adult staminal cells, named satellite cells, present in that muscle [5]. Satellite cells, quiescent under resting conditions, become activated, expand and differentiate during skeletal muscle regeneration in a process controlled by the expression of Pax genes and sequential expression of myogenic regulatory factors: MyoD, Myf5, Myogenin and MRF4 [6]. Satellite cell activation, proliferation, differentiation and subsequent fusion generate ex novo other multi-nucleated cells (myotubes) with characteristics similar to the fibers constituting the originating muscle. In addition, pluripotent cells capable of differentiating into the muscle phenotype are also present in other tissues, such as the heart, bone and, above all, the walls of vessels [7,8,9]. Several studies in different laboratories have tended to classify the protein factors derived from contractile activity as a subset within a more varied family not exclusively originating from skeletal muscle. In fact, many cytokines, such as myokines, can also be produced by other organs or tissues, such as bone or adipose tissue, and not all of them have a clearly identified systemic role or target organs other than muscle [10,11]. Several years ago, by comparing secretomes at different stages of differentiation processes in C2C12 cells (murine muscle cell line), about 635 secreted proteins, including 35 growth factors, 40 cytokines and 36 metallopeptidases, were identified [12]. Since then, the list of possible myokines has grown to over 3000, including those identified in the human species, such as angiopoietin, brain-derived neurotrophic factor (BDNF), fibroblast growth factor 21 (FGF21), myostatin (GDF8), nerve growth factor (NGF), S-100 proteins, a wide range of inflammation-related factors, such as interleukin-6 (IL-6), IL-7, IL-8 and IL-15, and the recently characterized irisin [13]. The large presence of these proteins, which can act as powerful mediators of signaling to other cells and tissues, highlights the important role of skeletal muscle as a prominent secretory organ. In humans, myokines released as a consequence of muscular contraction (and therefore, during physical activity) constitute a particular class known as “Exerkines”, which, by paracrine/endocrine means, are able to mediate beneficial effects throughout the body [1]. However, the synthesis and release of Exerkines as a result of physical exercise is not unique to the skeletal muscle, as they also reside in other organs and tissues. Thus, as a result of physical activity (not necessarily intense and/or of long duration), myokines can be secreted by skeletal muscles, adipokines can be released by adipose tissue and other factors can be secreted by the bones, liver and the brain and peripheral nervous system to then circulate in the body [14]. However, the molecular mechanisms that promote cross-talk between organs and organize the pro-metabolic and anti-aging effects of endurance exercise remain to be investigated. Since the extracellular milieu is presumably not a hospitable environment for labile Exerkines, a lipid vehicle-based mode of delivery has arisen over the course of evolution. In fact, physical activity can stimulate the secretion of two types of small membranous extracellular vesicles: exosomes (smallest extracellular vesicle, 20–140 nm, derived from inward budding of late endosomes that are released to the extracellular environment) and microvesicles or nanovesicles (large extracellular vesicles, 100–1000 nm, formed from the plasma membrane and released into the extracellular environment) [15]. Both types of delivery vehicles can carry proteins and/or nucleic acids and are involved in a variety of physiological and pathological processes. Exosomes, in particular, have been shown to facilitate the exchange of peptides, microRNA, mRNA and mitochondrial DNA between cells and tissues [16]. The composition of secreted vesicles depends, at least in part, on the type of exercise performed [17]. In sum, due to their ability to deliver useful molecules in different physiological and pathological conditions, extracellular vesicles may be promising candidates for potential therapeutic applications for different functional states, such as fragility due to aging, metabolic syndrome, some forms of neoplasia and more.

One of the most interesting scenarios to test this hypothesis is muscle ageing known as sarcopenia. Sarcopenia is the progressive loss of skeletal muscle mass, strength and/or correct function with aging, and is detrimental to human quality of life [18]. The causes of sarcopenia are generally attributable to natural aging processes, which are neither identified with sufficient certainty nor tested with adequate clarity. In practice, the only certainty in this respect is that aging processes are numerous and interlinked but lack a clear cause/effect relationship.

More solid evidence is available on the co-factors contributing to the development of sarcopenia. These include a decrease in the size and number of type II muscle fibers, a sedentary lifestyle, obesity, the presence of metabolic syndrome, reduced plasma concentrations of steroid hormones (androgens) and growth factors and a reduced muscle protein synthesis rate, even in the presence of protein meals or after endurance exercise [19].

The use of animal model organisms, such as mice, rats, flies and worms, has advanced the field of sarcopenia research, enabling the identification of some therapeutic strategies and/or dietary and lifestyle behaviors that result in improved muscle mass and function in old animals [20]. In rodents, aged flies and worms, dietary restriction improves muscle performance. In rodents and worms (but also in humans), exercise and a range of natural compounds alleviate the effect of muscle aging [21]. Reducing the insulin/IGF1 receptor pathway, well known to promote longevity, also improves sarcopenia [22]. In animal models, mitochondrial dysfunction (fragmentation and/or decreased number) may also contribute to the onset of sarcopenia: in rodents, there is an age-dependent reduction in mitochondrial mass associated with a change in morphology; in nematodes, there is an age-dependent fragmentation of mitochondria that precedes sarcomeric disorganization [23]. The lack of stem cells in worms and flies provides the opportunity to study processes that promote muscle maintenance without the confounding influence of muscle regeneration related to stem cell activity [20]. Information on the molecular mechanisms and structural changes that occur in this tissue with age originates from studies on muscle biopsies, including from humans.

It has been suggested that sarcopenia may be triggered by reactive oxygen species (ROS) that have accumulated throughout one’s lifetime. Many enzymatic and non-enzymatic antioxidant systems exist to eliminate excess ROS, prevent and repair the damage that they cause and maintain redox homeostasis in the cell. This is achieved in three distinct ways: (1) by converting ROS into less damaging molecules, (2) by reducing pro-oxidant molecules and (3) by activating ROS scavenging. These three systems can be interconverted according to cellular needs and can work synergistically to protect cells from accumulating oxidative damage [24]. Unfortunately, this homeostatic system tends to become less efficient with age, first in males and then in females, and in the skeletal muscle overall, ROS accumulate in the tissue, where these reactive elements are usually quite harmful and can damage other cellular components, such as DNA, contractile proteins and membrane lipids.

**Figure 1:**
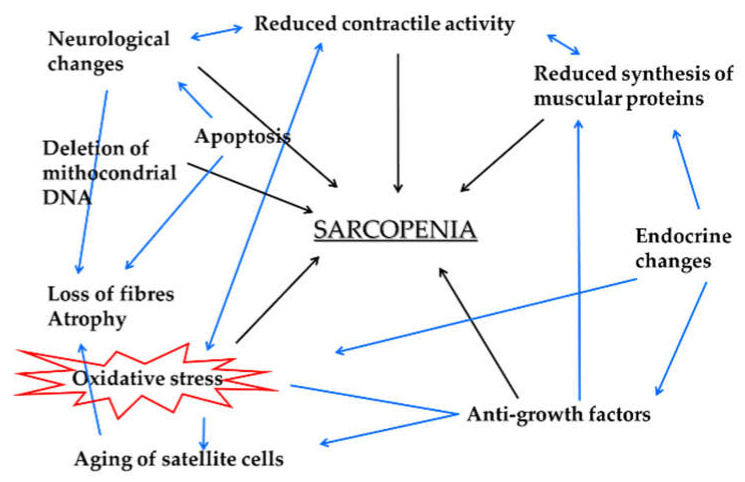
The role of oxidative stress in sarcopenia. Skeletal muscle aging is a complex process that is associated with a decrease in mass, strength and velocity of contraction, known as sarcopenia. This process is the result of many cellular changes. Notably, sarcopenia is triggered by reactive oxygen species (ROS), resulting in oxidative stress that can damage DNA, proteins, lipids, etc., causing further damage to the cells and tissues. Black arrows represent direct correlations with sarcopenia, while blue arrows represent indirect ones.

As a consequence of this damage, in the muscle fibers, the intra- and intercellular membrane networks, particularly those of the sarcoplasmic reticulum, may be modified, and the calcium transport mechanism may be altered [25]. This scenario could represent what happens in elderly muscle as a consequence of the altered function of the respiratory chain and of cellular antioxidant defenses. However, the various steps of the proposed mechanism are yet to be fully understood. In addition, the presence of oxidative stress in the skeletal muscle plays a significant role in the progression of sarcopenia because it leads to a substantial reduction in the regenerative potential of muscle fibers linked to the recruitment of quiescent satellite cells, as shown in Figure 1 [26].

Sarcopenia, however, is not an irreversible state, because physical exercise can reverse the process within some limits [27]. There is sufficient evidence that the release of some myokines from skeletal muscle can be increased following physical activity in both young and old people [28]. However, it remains to be established: (1) whether there is a direct interaction between myokines and antioxidant action and the identity of their intracellular target, (2) whether physical activity has the same effect in young and old subjects and the related intracellular pathways activated and (3) whether pharmacological and/or physical induction of muscle myokines is effective in reversing the oxidative stress process present in sarcopenic muscle.

During aging, the ability of skeletal muscle to sustain an efficient regenerative pathway is severely impaired, despite the presence of a reduced, even if apparently normal, pool of satellite cells [29]. Although definitive answers are lacking, recent experimental evidence suggests that the mere presence of endogenous stem cells may not be sufficient to ensure muscle regeneration and that the presence of appropriate stimuli and factors as well as protected environments is necessary to provide permissive conditions for stem cell-mediated regeneration [30].

On the basis of the premises that have been made, the attention was focused on the relationship between myokines present in the vesicles released by exocitosis by muscle cells and their possible anti-ageing effect on the metabolic processes mediated by the alteration of the oxidative state of senescent muscle.

In this review, we summarize our current knowledge on myokines, focusing on those that are present in the functional environment surrounding the muscle cell known as the “Niche”.

## 2. Features of Selected Myokines

The myokines characterized in this review were selected on the basis of two key criteria:

(1) The manifest ability of the myokine to act both from the inside of the cell and in an autocrine fashion;

(2) The existence of a definite relation between the presence of the myokine and the modulation of the ROS balance in fibers involved in regulatory processes (metabolic or regenerative) of muscle aging.

For this purpose, the following myokines were selected: Myostatin, IGF-1, NGF, S100 and irisin. In particular, in addition to being one of the first described myokines, Myostatin (Mstn) is undoubtedly a potent inhibitor of protein muscle synthesis (and is relatively important in the regenerative pathway). IGF-1 exerts a strong stimulatory effect on protein synthesis and energy availability in the muscle that secretes it and plays a leading role in the regenerative capacity of muscle fibers. NGF (which appears to be specific for skeletal muscle) is known for its particular action on regenerative capacity in muscle fibers. S-100, a little-known myokine, is capable of modulating contractile activity and force generation (but also acts on myogenesis), and finally, the extracellular level of irisin, which is not only the most recently discovered but also certainly one of the most interesting of the known myokines, is strongly linked to physical activity and is able to correlate the homeostatic effect on the secreting muscle with that on other systems (skeletal and cardiovascular systems in primis), forming a functional UNICUM of utmost importance for understanding the senescence process. Table 1 summarizes the principal information on the selected myokines.

### 2.1. Myostatin

The transforming growth factor-beta (TGF-beta) superfamily includes a group of growth factors directly involved in maintaining the homeostatic state of the organism. This family includes the first myokine defined as such in 1997 by McPherron et al., in mice: myostatin or growth and differentiation factor-8 (GDF-8), which is expressed in both embryonic and adult skeletal muscle. Myostatin is secreted by skeletal and cardiac muscle cells and acts locally to negatively modulate skeletal muscle mass [31]. The muscle-specific action of myostatin becomes evident when the gene controlling its expression is silenced: GDF-8-null mice are significantly larger than wild-type animals and have increased skeletal muscle mass that appears to be the result of both hyperplastic and hypertrophic activation of muscle cells. These results suggest that GDF-8 functions specifically as a negative regulator of skeletal muscle growth [32].

Myostatin is abundant in skeletal muscle, but it is also expressed in adipose tissue and heart muscle; it is widely conserved on the evolutionary scale, and the effect observed in mice is also found in dogs, sheep, cattle and humans [33]. However, attempts to apply the results obtained in animals to humans in order to test possible applications were rather disappointing [34].

Nevertheless, its biology is not as simple as it may appear. Myostatin and other members of the TGFβ family can both increase muscle growth and induce atrophy, depending on the downstream signaling that they activate. These factors bind to activin type IIA and IIB receptors (ActRIIA/B) and TGFβ receptors (TGFβRII) in the plasma membrane. They negatively regulate muscle mass by activating activin, which is a receptor-like kinase (ALK)-4, -7 and -5, which in turn phosphorylates SMAD2/3 and promotes the formation of a heterotrimeric complex with SMAD4 [35]. SMAD 2/3 can inhibit the transcription factor JunB, which normally promotes muscle growth and inhibits atrophy by blocking FoxO3 [36]. Although it is unclear how these factors regulate muscle mass, some evidence suggests that they affect the Akt/mTOR axis [37]. Despite the canonical TGF-β pathway inhibiting skeletal muscle growth and that it can enhance muscle atrophy, recently, researchers have found the parallel bone morphogenetic protein (BMP)-Smad1/5 signaling as an important positive regulator of muscle mass [38]. Consequently, multiple TGF-β family ligands can cooperate with, or counteract, myostatin activity, competing for the same receptor complexes and Smad-signaling proteins [39].

When Myostatin acts on the whole cellular apparatus of the muscle through the receptor ActRII/B, the intracellular domain of the ligand–receptor complex forms a serine/threonine kinase-based complex that is transferred to the nucleus to regulate the transcription of genes involved in the proliferation and differentiation of skeletal muscle stem cells. In mature fibers, Myostatin not only activates the protein degradation pathway but also, in mammals, inhibits the positive modulation system of protein synthesis mediated by mTOR in response to growth signals such as insulin and IGF-1. The final result of myostatin action is a reduction in muscle trophism, with a reduced ability to restore the skeletal muscle tissue via satellite cell activation [40]. Indeed, Myostatin has been shown to play an important role in skeletal muscle wasting by increasing protein degradation, as occurs in aging. Myostatin may be considered a pro-oxidant and appears to induce oxidative stress by producing ROS in skeletal muscle cells through tumor necrosis factor-α (TNF-α) signaling via NF-κB and NADPH oxidase. Aged Mstn-null (Mstn^−/−^) muscles, which have reduced sarcopenia, also contain increased basal antioxidant enzyme levels and lower NF-κB levels, indicating efficient scavenging of excess ROS. For this reason, the inhibition of Mstn-induced ROS could lead to reduced muscle wasting during sarcopenia [41].

As mentioned above, the role played by Myostatin has also been demonstrated by experiments carried out with knockout animals for the *myostatin* gene, in which both hypertrophy and skeletal muscle hyperplasia can be detected. These cellular adaptations produce a hyper-muscular phenotype in several species, including humans [42]. While myostatin may be the best-known member of the TGFβ superfamily, this family of growth factors consists of at least thirty elements. Among these, growth differentiation factor 11 (GDF11) deserves special attention. GDF11 was initially thought to mimic the action of myostatin. Although there is much overlap between the two proteins in terms of both amino acid sequence and receptor and signaling pathways, accumulating evidence suggests that these two ligands have distinct functions [43]. GDF11 appears to be essential for normal mammalian development and has recently been proposed as an active regulator of tissue aging [44]. Myostatin, on the other hand, appears to have a suppressive effect on skeletal (and cardiac) muscle mass through negative regulation of cellular metabolic processes. It should be noted that these effects occur not only in muscle but also in the brain [45].

The pathophysiology of sarcopenia is multifactorial, with the constant presence of intracellular oxidative stress associated with hormonal decline and increased myostatin signaling, which are closely associated with muscle dysfunction followed by atrophy. In vitro experiments show that exposing muscle cells to H_2_O_2_ induced abundant intracellular ROS production and mitochondrial dysfunction and increased myostatin expression through nuclear factor-κB (NF-κB) signaling [46]. In aged skeletal muscle, inflammation and oxidative stress appear when specific regulatory molecules associated with wasting are activated (such as the ubiquitin–proteasome system and myostatin) or repressed (e.g., IGF-1 and PGC-1α).

Currently, therapeutic interventions based on decreasing myostatin levels have not been established to successfully treat muscle wasting. Exercise, however, is an effective stimulus that can attenuate the imbalance between protein synthesis and degradation, thus restoring at least part of the muscle’s functional capacity [47].

### 2.2. NGF

Neurotrophins are a family of growth factors that regulate the trophism, differentiation and plasticity of nerve cells. According to most opinions, this family consists of nerve growth factor (NGF), brain-derived neurotrophic factor (BDNF), neurotrophin-3 (NT-3) and neurotrophin-4 (NT-4). Their signaling to target cells begins with binding to two receptor classes: the three types of tropomyosin-related kinase receptors (Trk A-B-C) and the unique p75 neurotrophin receptor (p75NTR).

The receptor–ligand system is partly specific because NGF shows a high affinity for TrkA, and NT-3 activates TrkB, while both BDNF and NT-4 preferentially bind to TrkC. In contrast, there appears to be no selectivity for the p75NTR receptor, which binds all neurotrophins with low affinity [48].

The fact that protein factors are contained in and secreted from skeletal muscle was proven in the mid-1980s when the presence of “an active factor that is heat labile, trypsin sensitive, and non-dialyzable, and it has negligible neurotrophic effect” was demonstrated in skeletal and cardiac muscle [49].

A few years later, this factor was better defined, and it was found to be identifiable as NGF, normally produced and secreted by the nervous system [50]. In particular, it was found that the levels of NGF in rat heart muscle were significantly higher than in skeletal muscle, but a cause/effect relationship based on age between the muscle concentration of the growth factor and the trophic state of the examined animal was not established [51]. In addition to the known roles played at the level of the nervous system, experimental data indicate that neurotrophins (in particular, NGF) are involved in muscle regeneration. Indeed, NGF improved the muscle-regenerating capacity of muscle stem cells in dystrophic muscle [52]. The skeletal muscle tissue synthesizes and secretes NGF [53], and its expression and its p75NTR receptor in myoblasts are developmentally regulated during myogenesis [54]. In addition, phenotypic knockout of NGF resulted in skeletal muscle atrophy and dystrophy in adult mice. In humans, regenerating muscle fibers from patients affected by Duchenne and Becker muscular dystrophies consistently express NGF, as do myofibroblasts and mast cells. This effect can be produced by NGF released directly from muscle fibers and/or muscle stem cells. Indeed, Ettinger et al. demonstrated that C2C12 myoblasts, a mouse skeletal muscle myoblast cellular model, secreted NGF to the media by playing an autocrine proliferative role, whereas it was not secreted by C2C12 myotubes [55].

The myogenic satellite cell has an anatomically defined specialized niche that ultimately governs the state of this cell population (quiescence, activation, proliferation, etc.). The adjacent differentiated myofiber, innervating motor neurons, infiltrating inflammatory cells and vascularization collectively establish the niche in which the satellite cell resides [56]. Released cytokines, neurotrophic factors, growth factors and oxygen tension, such as Hif1α, Hif2α, NO and Vegf, collectively orchestrate and modulate the status of the satellite cell pool. During muscle development or regeneration, myocytes transiently produce NGF, as well as its tyrosine-kinase and p75 receptors, but when myoblasts were screened for the expression of NGF receptors, only p75NTR was detected, while the high-affinity NGF receptor, TrkA, was not present [57]. Recent studies have suggested that NGF stimulates myoblast differentiation and collagen synthesis, but the regulatory mechanism remains poorly defined [58]. In addition, it has been reported that the p75NTR receptor could represent a key regulator of the NGF-mediated myoprotective effect on satellite cells, but the precise function of the NGF/p75 signaling pathway in myogenic cell proliferation, survival and differentiation remains fragmented and controversial [59]. Using myoblasts as a substrate, a relationship has been shown to exist between NGF and the type of muscle fiber formed at the end of the differentiation process. Specifically, the proNGF/p75NTR pathway facilitates a slow-to-fast fiber type transition by counteracting the expression of slow myosin heavy chain. Simultaneously, activation of proNGF/p75NTR facilitates the induction of fast myosin heavy chain [60].

However, the effect of NGF on muscle is also expressed through direct actions on tissue trophism because, in mice subjected to thermal stress for different periods of time, a direct correlation between increased expression of the *NGF* gene and the protective effect on muscle tissue has been demonstrated [61]. Furthermore, when C2C12 cells undergo a reoxygenation insult, they have a more oxidized redox potential following the generation of reactive oxygen species (ROS). Ettinger et al. (2012) showed that the presence of βNGF during reoxygenation determines the maximum myoprotective effect in C2C12 myotubes. The authors hypothesized that, similar to βNGF, NGF induces the rapid activation of the antioxidant defense systems, lowering the level of ROS. They also hypothesized that, under stress conditions, the level of NGF increases with consequent autocrine activation of the muscle and increased survival/myoprotection [55].

In this regard, some data obtained in rats suggest that endurance exercise (10 days of treadmill exercise) can also increase skeletal muscle mass and intramuscular NGF concentrations, at least in experimental autoimmune encephalomyelitis. In these animals, physical activity led to a significantly greater bilateral increase in EDL, plantaris and gastrocnemius muscle mass than in sedentary controls. The same muscles had significantly higher NGF concentrations relative to the controls [62]. Interestingly, higher expression of various neurotrophins (including NGF) and the p75NTR receptor was observed in muscle progenitors obtained from presomitic extraocular muscles compared to somitic muscles. Extraocular muscles exhibit greater resistance to muscular dystrophies and sarcopenia [63]. They were recently shown to have different types of myogenic cells, all of which have exceptional regenerative potential. Neurotrophins are important modulators of myogenic regeneration and act by promoting the proliferation of myoblasts, improving myogenic fusion rates and protecting myotubes from stress stimuli, including oxidative stress. As a result, these muscles are better protected against stress and sarcopenia [64].

In fact, data published several years ago suggest that the age-related decline in skeletal muscle mass is not linked to reduced local NGF concentrations; such a cause/effect relationship with NGF concentration is not evident in skeletal muscle [51]. However, NGF stimulation significantly enhanced the engraftment efficiency of adult staminal cells transplanted in the dystrophic muscle of mdx mice, resulting in the regeneration of numerous dystrophin-positive muscle fibers [52]. In a mouse model of hindlimb ischemia, *NGF* gene transfection could enhance the expression of its protein, and this induced an increase in the presence of type I muscle fibers. On the contrary, no measurements have been made to verify the correlation between the state of oxidative stress that results from ischemic atrophy and myokine transfection [65]. NGF is critical for neuronal differentiation and maintenance through the activation of TrkA and p75 receptors. In particular, mitochondria play a crucial role during neurogenesis and in post-mitotic neurons, providing the energy required for neuronal activity and synaptic function. Several studies have found that neuronal differentiation is accompanied by metabolic reprogramming to meet increased energy demand [66]. This is achieved by promoting oxidative phosphorylation, leading to increased ROS generation and the need to increase mitochondrial biogenesis, along with control of mitophagy mechanisms [67]. With a few minor modifications, the framework established for the neuron could also be used to describe mechanisms in the muscle fiber. Here, too, the increased energy requirements of contractile function lead to an increase in oxidative phosphorylation and ROS generation and the need to act on both mitochondrial biogenesis and mitophagy. However, no data have been published on this issue, and the statement is, therefore, only a working hypothesis.

### 2.3. IGF-1

In the long list of myokines that are not exclusive to muscle tissue, insulin-like factors deserve separate but thorough consideration.

The insulin-like growth factor (IGF) system stimulates growth and proliferation and regulates cell differentiation in a tissue-specific manner. The system is composed of two insulin-like growth factors (IGF-1 and IGF-2), six insulin-like growth factor-binding proteins (IGFBPs) and two insulin-like growth factor receptors (IGF-1R and IGF-2R). IGF actions occur mostly through the activation of plasma membrane-bound IGF-Rs by circulating ligands (IGFs) released from the IGFBPs that stabilize their levels in the serum [68].

Since the discovery of the presence of insulin-like growth factors in the mid-1950s, the amount of information accumulated has been able to provide a unique scenario for insulin and IGFs, which could be considered a single composite family of hormones with similar molecular nature and properties (insulin and IGFs), cell surface receptors (IR, IGFRs) and other accessory components (IGFBPs) that, in a highly coordinated and synergistic manner, regulate multiple biological processes [69]. This suggested the hypothesis that there is a “division of labor” between insulin, IGF ligands and receptors. This resulted in the classic dogma that prevailed for many years in the field, which attributed a mainly metabolic role to the insulin/IR system and a mitogenic, proliferative/differentiative role to the IGF/IGFR system [70]. However, new information generated in recent years has clearly shown that many of these ‘‘old’’ concepts, although correct in terms of their basic assumptions, are oversimplifications of much more complex situations.

In this regard, at least two other considerations need to be addressed to better understand the complex interaction linking the biological effects of insulin and IGFs. The first is that the evidence accumulated in recent years indicates that insulin-R and IGF-1R are present in the nucleus (but also in the Golgi apparatus) of both normal and transformed cells, and thus, display a range of overlapping activities that fall under the definition of transcription factors [71]. The second situation arises when the activities of the IGF and insulin chain are dysregulated in the so-called insulin/IGF resistance state. The term “insulin/IGF resistance” describes a phenomenon in which the body exhibits blunted activation of the IR and IGF1R signaling cascades. To counteract this resistance, beta cells increase the production and secretion of insulin to propagate sufficient insulin signaling, which can lead to hyperinsulinemia. Under these conditions, a strong integration of the insulin/IGF signaling pathways and the second messenger ROS-mediated redox signaling pathways involve regulatory cross-talk processes between these pathways [72]. In particular, in the skeletal muscle, the insulin resistance induced by elevated plasma free fatty acid plays an important role in the development of insulin resistance in this tissue [73].

In general, the IGF receptor displays the characteristics of a tyrosine kinase receptor, as the phosphorylation of tyrosine residues induces its biological effects. However, more recent research showed that IGF-IRs also exhibit kinase-independent functions and can also activate signaling cascades through non-canonical pathways that are not fully known. Finally, IGF-IRs have a broad spectrum of cross-talk with many other tyrosine kinase receptors [74].

In human skeletal muscle, the gene encoding IGF-1 is capable of structuring multiple heterogeneous mRNA transcripts through a combination of different transcription sites and alternative splicing. These transcripts encode different isoforms of the IGF-1 precursor peptide, such as IGF-1Ea, IGF-1Eb and IGF-1Ec, also known as mechanical growth factor or MGF when present in skeletal muscle. In addition, all of these isoforms can undergo post-translational modifications. The identification of locally expressed and load- or damage-sensitive IGF-1 isoforms in skeletal muscle has been one of the most interesting developments in the context of the autocrine/paracrine actions of IGF-1 because the mechanisms underlying the different actions of this agent on skeletal muscle trophism and/or activity could be regulated by specific IGF-1 isoforms [75]. Insulin-like growth factors are key factors in the regulation of both anabolic and catabolic pathways in skeletal muscle. In particular, IGF-1 stimulates protein synthesis in skeletal muscle via the PI3K/Akt/mTOR and PI3K/Akt/GSK3β pathways. The former pathway can also inhibit FoxOs and protein degradation mediated by the ubiquitin–proteasome system (UPS). Autophagy mediated by mTOR and FoxO signaling also appears to be regulated, at least in part, by IGF-1 [76].

IGF-1 also enhances skeletal muscle regeneration through the activation of satellite cells, thus resulting in a stimulus for hyperplasia. Importantly, IGF-1 levels and downstream IGF-1R signaling are suppressed in many chronic disease conditions, such as cachexia and fibrosis [77]. Finally, in the opinion of many, IGF-1, Akt/Protein Kinase B and the target signaling pathway mTOR constitute the key link between muscle contraction and protein synthesis in its fibers. If this is true, then the alteration of the pathway described above could lead to sarcopenia [78].

Specifically, activation of mTOR is a consequence of the role that insulin and IGF-1 play synergistically in controlling muscle mass. IGF-1 and insulin act by binding to their respective receptors, and this triggers the activation of several downstream kinases, culminating in the activation of Akt [79].

During muscle atrophy, decreased binding of IGF-1 and/or insulin to their respective receptors and/or increased binding of glucocorticoids to the glucocorticoid receptor results in reduced activation of Akt/mTOR. This leads to a decrease in protein synthesis. Decreased mTOR activity also leads to the stimulation of autophagy through ULK1/2 signaling [80].

At the same time, reduced Akt activity causes the release of FoxO from segregation sites in the cytoplasm, and this triggers an atrophic cascade linked to the expression of atrogenes belonging to the proteolytic pathways of lysosomal autophagy and the ubiquitin cycle in the proteasome [37].

Furthermore, hyperactivation of the autophagy mechanism increases muscle atrophy, as induced by many physiopathological conditions. These include cachexia, fasting, disuse and oxidative stress, as demonstrated in a mouse model of amyotrophic lateral sclerosis (ALS) with a mutation in superoxide dismutase (SOD1G93A) [81].

In this regard, in a literature review published in Frontiers in Nutrition, Richie D. Barclay et al. proposed the definition of some functional metabolic parameters that make the role of IGF-1 in managing the muscle aging process more understandable. Barclay stated: “Human skeletal muscle is highly plastic and is in a constant state of remodelling. Skeletal muscle remodelling occurs due to the dynamic balance between muscle protein synthesis (MPS) and muscle protein degradation rates (MPB). The daily difference between MPS and MPB defines the net protein balance (NPB), which is a key regulator of overall skeletal muscle mass. A positive NPB is generally indicative of a positive remodelling response that may be hypertrophic (i.e., increase fibre cross-sectional area) or non-hypertrophic (i.e., increase metabolic quality) in nature, whereas a reduced NPB reflects an obvious phenotype of being negative by inducing a loss of muscle mass or poor metabolic quality. Changes in MPB are small in normal aging, whereas changes in MPS appear to be larger in magnitude and more apparent in response to major anabolic stimuli to muscle tissue. As such, measurement of MPS is the primary goal in human metabolic research” [82].

Physical activity is considered one of the main strategies to counteract muscle decline in the elderly. Exercise reduces age-related oxidative damage and chronic inflammation, stabilizes autophagy processes and improves mitochondrial function. It also improves myokines, at least exerkines, and the IGF-1 signaling pathway [83]. In particular, IGF-1 mediates a protective mitochondrial signal that is transduced into the cell through the transcription factor nuclear factor erythroid 2-related factor 2 (Nrf2). By coupling mitochondrial biogenesis with the induction of BNIP3 (a member of the apoptotic Bcl-2 protein family), this pathway increases autophagosome turnover and improves cell survival, even in the presence of metabolic or mitochondrial stress [84]. In recent years, IGF-1 signaling has been shown to modify mitochondrial function and capacity (including mitochondrial DNA/RNA ratio management), organelle biogenesis, oxidative phosphorylation and suppression of ROS production [85]. In addition, IGF-1 has been proposed and tested as a therapeutic agent (in low doses) capable of inducing several beneficial effects, such as a reduction in insulin resistance and a significant improvement in lipid dysmetabolism. For these reasons, IGF-1 therapy has been able to exert clear mitochondrial protective effects and antioxidant and neuroprotective effects [86].

### 2.4. S-100

Another myokine family that we discuss in more detail is composed of a series of small proteins with a canonical weight of 10,000 D, discovered in the bovine brain in the second half of the 20th century, named S100 [87]. These proteins form an important subclass of EF-hand calcium-binding proteins, are highly conserved on the evolutionary scale, and are specifically expressed in different tissues and cells, a feature that they share with most other EF-hand Ca^2+^-binding proteins, such as troponin and calmodulin [88]. The S100 protein is effective either as a monomer or as a dimer. The S100 family consists of more than twenty members distributed in three groups: (a) those with intracellular regulatory activity, (b) those with intracellular and extracellular functions and (c) components whose functional effects occur extracellularly [89].

With regard to intracellular action, S100 proteins are involved in aspects of proliferation/differentiation regulation, Ca^2+^ homeostasis, energy metabolism and inflammation through interactions with a variety of target proteins, including enzymes, receptors, transcription factors and others [90].

Many members of the S100 family are secreted and regulate cellular functions in an autocrine and paracrine manner through the activation of surface receptors (e.g., RAGE) or G-protein-coupled receptors, scavenger receptors and *N*-glycans [91].

Thus, extracellular S100 proteins exert regulatory activities on white cells of the inflammatory process, endothelial and vascular smooth muscle cells, nervous system cells, skeletal muscle fibers, myoblasts and cardiomyocytes. Thus, S100, with its various modalities, participates in immune responses, cell migration, tissue development and repair and tumor cell invasion [92].

Adult muscle tissues contain high levels of S100 protein, but the particular form present depends on the type of muscle: cardiac muscle exclusively contains S100A, slow-twitch skeletal muscle fibers predominantly contain S100A, vascular smooth muscle contains both S100A and S100B and fast-twitch skeletal muscle fibers contain low but detectable levels of S100A and S100B [93]. In skeletal muscle, the protein has been shown to colocalize with structures involved in excitation–contraction coupling [94].

In mammalian skeletal muscle, contraction occurs because the intracellular Ca^2+^ concentration increases by about 100 times compared to that at rest. The ionic increase occurs due to release mediated by a specific channel (RyR) located on the sarcoplasmic reticulum, which is regulated physiologically by the potential that propagates during muscle excitation [95].

The functional state of the RyR (closed, open, inactivated) largely depends on the intracellular calcium concentration and the state of oxidation of its protein components at particular sites [96]. Today, experimental evidence seems to indicate that there is a mechanism in skeletal muscle that can finely modulate the functional status of the RyR channel and, thus, the available Ca^2+^ required for contraction. This fine-tuning occurs at specific common RyR sites through interaction with two calcium-binding regulatory proteins present in the sarcoplasm and reticulum membranes, respectively: Calmodulin and S100A [97]. However, fundamental information necessary for a detailed description of the molecular dynamics of this interaction is still lacking [98].

The autocrine/paracrine capacity of secreted S100 could result from its internalization by a membrane vesicle formation mechanism [99], mediated or not by the ligand–receptor complex binding to RAGE, whose modulation is fundamental in several mechanisms in the skeletal muscle, such as the recruitment and the maturation of precursors in both development and postnatal regenerative phases [100]. On the other hand, dysregulated RAGE activity in adult skeletal muscle is a feature of muscle wasting that occurs in aging [101].

The S100 protein is effective as either a monomer or a dimer [102]. In this regard, it has been reported that Calprotectin, a myeloid-related inflammatory protein, also known as MRP8/14, is a heterodimer composed of two intracellular calcium-binding proteins, S100A8 and S100A9, expressed not only in muscle but also in human neutrophils, monocytes and macrophages. Elevated plasma levels of calprotectin have been reported in a variety of chronic inflammatory conditions, including rheumatoid arthritis, inflammatory bowel disease, cancer and COVID-19 disease [103,104].

It was hypothesized that the synthesis and secretion of other factors by the muscle, including calprotectin, might be induced by IL-6, a cytokine secreted by skeletal muscle, in an autocrine or paracrine fashion. Microarray analysis performed on human muscle biopsies obtained up to six hours after IL-6 infusion identified the dysregulation of a small set of genes. The use of RT-PCR confirmed that S100A8 and S100A9 mRNA were up-regulated three-fold in skeletal muscle after IL-6 infusion compared to controls. In contrast, a five-fold up-regulation of S100A8 and S100A9 mRNA was recorded after 3 h ergometer exercise in healthy young males. Under these experimental conditions, plasma calprotectin increased five-fold. In contrast, no increased secretory activity was recorded after just IL-6 infusion. These data strongly indicate that calprotectin secretion from the skeletal muscle of young men is a consequence of physical activity and not of pro-inflammatory inducers such as IL-6 [105].

As mentioned above, many members of the S100 family (mainly A and B) exert both intracellular and extracellular effects [106]. In the last decade, many studies have provided more detailed information on the mechanisms of action of S100B as an intracellular regulator and extracellular signal. Within cells, S100 proteins are involved in many aspects of functional activity, such as regulation of the cell cycle and mechanisms controlling cell differentiation and death. Many members of the S100 family are secreted and regulate cellular functions in an autocrine and paracrine manner through the activation of surface receptors [89]. If this is also true for muscle cells, then S100 may not only have the previously described role in the modulation of RYR but also intervene in one of the key processes of muscle senescence: the regenerative capacity of staminal cells. Satellite cells of sarcopenic muscle and proliferating aged myoblasts accumulate ROS due to altered mitochondrial homeostasis and impaired antioxidant systems [107]. Among other detrimental effects, ROS imbalance can adversely affect the autophagy mechanism, which could be one of the main contributors to the negative changes in the proliferative and differentiative capacity of aged muscle stem cells [108]. The presence of S100B in relatively high concentrations in all types of satellite cells (quiescent, proliferating myoblasts, myotubes) and myofibers suggests the possibility that S100 is involved in the regulation of cellular processes that lead to muscle regeneration because it is constitutively expressed in quiescent SCs, proliferating myoblasts, myotubes and myofibers [109]. S100B is passively released from injured muscle tissue, and high levels of S100B are also detectable in human plasma after intense exercise [110,111]. The myoblasts of sarcopenic subjects release relatively low amounts of S100B, so it could be hypothesized that the high levels of ROS in these cells alter the mechanism of S100B secretion and/or oxidize S100B, which is not secreted and accumulates internally. As a consequence of this, ROS overproduction in myoblasts causes S100B accumulation and stimulates NF-κB activity, which causes S100B up-regulation. In turn, S100B stimulates NF-κB activity, resulting in the transition of myoblasts into brown adipocytes [112]. One of the molecular mechanisms by which this is achieved involves both apoptosis and autophagy. Available data indicate that cell death promoted by S100A8/A9 occurs through cross-talk of mitochondria and lysosomes via ROS and BNIP3 [113]. In addition, S100A9 has also been shown to promote cellular senescence of bone marrow stromal cells via the TLR4/NLRP3 pathway and IL-1β secretion [114].

### 2.5. Irisin

In 2012, B. Spiegelman’s team (in collaboration with others) at the Dana-Farber Cancer Institute and Harvard Medical School, Boston, in a well-known article in Nature, described the discovery of a new myokine synthesized by skeletal muscle and secreted following mild physical activity, with an impressive ability to transform white fat into brown fat and increase glucose uptake by the muscle. Furthermore, shortly after the detection of irisin, data derived from mice were compared to those obtained in humans and found to be 100% overlapping, making it immediately clear that this myokine was a pivotal discovery. As a previously undescribed messenger from muscle to other tissues, the new polypeptide was named irisin, in honor of Iris, the Greek goddess messenger of the gods and personification of the rainbow [115].

Irisin is a myokine that is secreted by muscle cells expressing the transcription factor peroxisome proliferator-activated receptor-γ co-activator 1α (PGC1α), which is involved in many pathways related to energy metabolism. PGC1α stimulates the synthesis of the transmembrane protein FNDC5, whose protein sequence comprises a signal peptide, a fibronectin III domain, a hydrophobic transmembrane domain and a carboxy-terminal domain located in the cytoplasm. After proteolytic cleavage, a new protein consisting largely of fibronectin domain III is released. This protein, which consists of 112 amino acids, is irisin; its amino acid sequence is identical in humans and mice [115]. Notably, PGC1α transcription is controlled by cAMP via the CREB protein. Indeed, a direct link between exercise, cAMP and increased CREB levels, which in turn induces FNDC5 expression, has been identified. If CREB is inhibited, FNDC5 transcription is suppressed [116]. In addition, studies in C2C12 muscle cells also demonstrated that the cAMP-activated protein kinase pathway is involved in PGC1α/irisin expression [7]. In more recent experimental clinical studies in humans, a direct association between Sirt-1 levels and PGC1α/irisin expression was found in obese patients or those with type 2 diabetes [117]. The protein in the skeletal muscle is present as either a homodimer or a dimer formed by a β-sheet between Arg75 and Glu79 aa, which in turn protects the ends of the molecule and thereby stabilizes the structure [118]. The final detail to consider is that irisin is glycosylated before being secreted to preserve its biological and functional capacity [119].

The receptor for irisin has not been identified, and its effects remain uncertain, especially with regard to its autocrine action. The only data related to the presence of specific myokine binding sites were obtained from experiments on bone cells, one of the primary targets for irisin and a likely paracrine site of its action. Indeed, irisin has been shown to bind to proteins of the αV class of integrins, and biophysical studies have identified interaction surfaces between irisin and αV/β5 integrins. Furthermore, pharmacological inhibition of αV integrins blocks irisin signaling in osteocytes and fat cells [120].

At least one of the intracellular mechanisms by which irisin acts in the target cell is linked to the active regulation of cellular autophagy processes. Autophagy is a crucial step in the homeostasis of nutrients and energy in the cell and largely acts on cellular catabolism by facilitating lysosomal degradation and promoting the recovery and reuse of damaged proteins and organelles. This process is well conserved on the evolutionary scale and is naturally activated in response to states of nutritional deprivation and/or by the presence of pathogens. In conclusion, autophagy acts as a protective mechanism that allows cells to survive under stress conditions [121]. Irisin is widely distributed in the human body and is involved in metabolic processes such as the transformation from white to brown adipocytes and the mechanism of insulin resistance [122]. However, irisin also appears to have a positive effect on cognitive function and to play a role in bone metabolism regulation [2,123]. Finally, recent studies have shown that malignant cells have a higher concentration of irisin than normal ones [124]. Irisin function is apparently contradictory because it is able to induce both cytoprotection and cell death in a plasma concentration-dependent manner.

In humans, FNDC5 mRNA is mainly expressed in muscle and other muscle-containing organs, such as the pericardium and vessel walls, and it has also been detected in blood, saliva and cerebrospinal fluid [125,126,127]. Skeletal muscle (40% of body weight) is the main reservoir of irisin and, therefore, determines its circulating levels. Age-related loss of muscle mass can lead to lower circulating irisin levels in the elderly [125]. Indeed, recently, Chang et al. (2017) considered low serum irisin concentration as a sensitive molecular marker for muscle weakness and wasting and Park et al. (2018) proposed that in postmenopausal women, the decrease of blood irisin concentration is an independent predictor of sarcopenia [128,129].

Conversely, it has also been shown that circulating irisin levels increase with increasing fat mass, particularly in obesity. A large number of studies have shown that irisin has a potential role in certain metabolic diseases, such as diabetes and obesity, and is involved in the regulation of energy metabolism. For example, it increases thermogenesis, reduces lipid accumulation and maintains glucose homeostasis in skeletal muscle and other organs [130]. Abnormal glucose and lipid metabolism, diabetes and obesity are risk factors for cardiovascular disease, so irisin has a potential role in maintaining cardiovascular homeostasis [131].

Recent studies have suggested that irisin increases mitochondrial function in cardiomyoblasts and protects against ischemic and reperfusion injury in the murine heart ex vivo. In humans, however, it appears that acute myocardial infarction patients with elevated serum irisin concentrations are associated with a higher rate of adverse cardiovascular events. Based on clinical observations, some authors have hypothesized that an excess of irisin could lead to mitochondrial dysfunction and cardiomyocyte damage. In summary, increased expression of irisin in the heart and/or irisin treatment in cardiomyocytes increased ROS production, resulting in caspase-9-dependent apoptotic processes [132]. In effect, despite the efforts of various researchers, whether irisin protects the heart against myocardial ischemia and reperfusion injury (I/R) is still unknown. In experiments in which isolated hearts were subjected to 30 min ischemia followed by 30 min reperfusion, irisin treatment led to a marked reduction in the size of the myocardial infarction. In particular, irisin treatment increased SOD-1 and p38 phosphorylation but suppressed levels of active caspase-3 and annexin V [133].

In cardiomyoblasts exposed to hypoxia/reoxygenation, irisin treatment significantly attenuated hypoxia/reoxygenation, as indicated by the reduction in LDH loss and apoptotic cardiomyocytes. Furthermore, irisin treatment suppressed mitochondrial swelling and protected mitochondria function [134]. This hypothesis is supported by both in vivo and in vitro experiments that showed that GTPase OPA1, which is responsible for the regulation of mitochondrial dynamics and is crucial for adapting mitochondrial function and preserving cellular health, is downregulated in the infarcted heart, whereas irisin treatment upregulated its expression and protected cardiomyocytes from further damage after myocardial infarction [135]. Collectively, these results seem to indicate that irisin serves as a novel approach to elicit cardioprotection, which is associated with improved mitochondrial function [136]. Furthermore, serum irisin concentrations are reported to be inversely associated with the prevalence of coronary artery calcification after adjustment for age and behavioral factors. After adjustment for cardiometabolic risk factors, the inverse association between serum irisin concentration and coronary artery calcification progression persisted [137]. This suggests that circulating irisin concentrations have a potential role in predicting the onset and development of coronary pathology [138]. Similarly, another study found that in a sedentary rather than active lifestyle, circulating irisin concentrations were positively correlated with cardiovascular risk factors such as fasting insulin and fasting triglycerides [139]. However, it must be recalled that muscle mass is an important factor influencing circulating irisin concentrations, and the difference in muscle mass between subjects may also be a reason for the divergence in research results. As abnormal glucose and lipid metabolism, diabetes and obesity are risk factors for cardiovascular disease, irisin, which is an important regulator of energy metabolism, could play a key role in maintaining a healthy cardiovascular system [140].

Finally, atherosclerosis is characterized by the accumulation of lipids on the walls of arteries, which can lead to stenosis and arterial failure [141]. Circulating irisin concentrations have been shown to be negatively correlated with atherosclerosis parameters [142], and PGC1α is reported to be overexpressed in skeletal muscle; this leads to an increase in irisin secretion and reduces the atherosclerotic plaque area by 40% in mice [143].

Taken together, these data (and others not described here) from animal and human experiments, using both in vitro and in vivo approaches, appear to confirm that irisin is involved in the regulation of cardiovascular homeostasis. This result is the consequence of the action of this myokine on a complex package of molecular pathways, including ROS/NO production, autophagy, angiogenesis and inflammation in cardiomyocytes or vessel endothelial cells.

With regard to the autocrine action of irisin on muscle secretion, the currently available data seem to indicate that the main effect of the myokine is on glucose metabolism, energy utilization and availability and regulation of mitochondrial number and capacity [144].

Treatment of human myocytes with recombinant irisin significantly increases glucose and free fatty acid uptake, similar to that induced by insulin. This is a consequence of increasing the expression of genes that are involved in glucose transport and lipid metabolism in myocytes (GLUT4, HK2 and PPARA) by at least 30%. Conversely, the expression of genes that are involved in glycogenolysis (PYGM) or gluconeogenesis (PCK1) is drastically inhibited [145].

Irisin, even at lower concentrations (5 nM), stimulates mitochondrial biogenesis by increasing the gene expression of *Tfam, Ppargc1a and Nrf1*, as well as gene and protein levels of UCP3 and GLUT4, in murine C2C12 cells. Furthermore, irisin does not activate the nuclear factor-κB (NF-κB) pathway in C2C12 cells, indicating that this myokine, in contrast to TNF, may not be involved in inflammatory responses in muscle [146].

In skeletal muscle, PGC-1α, encoded by the *PPARGC1A* gene, plays an important role in the regulation of mitochondrial biogenesis and adaptation to aerobic training. Acute exercise activates PGC-1α, thereby modulating the transcriptional activity of its partners and regulating the expression of genes involved in mitochondrial biogenesis, angiogenesis and fat and carbohydrate metabolism [147].

In the first years after irisin was discovered, several studies examined the effect of exercise on irisin secretion and reported negative results. For example, in vitro exercise-mimicking treatment with forskolin and ionomycin in primary human muscle cell cultures stimulated PGC1α expression but decreased FNDC5 expression and irisin secretion [148]. Similarly, in vitro contraction of human skeletal muscle cells by electrical pulse stimulation increased PPARGC1A mRNA levels but had no effect on FNDC5 mRNA levels [149]. Some in vivo studies using different physical exercise protocols have also failed to detect an association between levels of irisin or PGC1α and exercise [150].

However, many other animal and human studies have shown an increase in circulating levels of irisin after exercise. For example, some investigators observed that irisin levels increased from 3.6 to 4.3 ng/mL in the serum after 12 weeks of high-intensity aerobic training in humans [151].

In mice, the level of irisin detected with western blotting was also 2-fold higher in skeletal muscle and 1.5-fold higher in serum after one bout of treadmill exercise. Immunohistochemical analysis showed that irisin was located extracellularly between muscle fibers [152].

**Figure 2:**
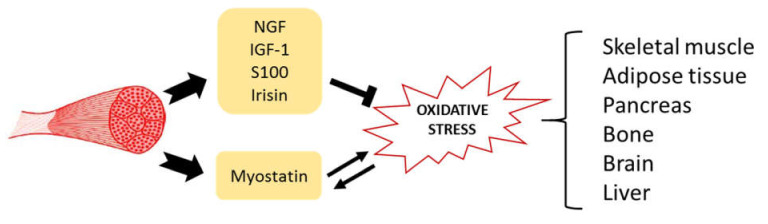
The role of myokines. Myokines are product of the muscle secretome; their action is widespread throughout the body. Most myokines are able to act specifically against oxidative stress, improving mitochondrial function and reducing ROS production, while myostatin increases oxidative stress that in turn increases myostatin itself.

PGC1α is highly expressed in tissues with high oxidative capacity and acts as a key metabolic regulatory factor in many physiological situations involving muscle, such as endurance programs and the resulting change in the ratio of fast-to-slow fibers that are often associated with changes in insulin sensitivity. PGC1α is both a cause and an effect of oxidative stress: its expression correlates directly with oxidative stress, but it is also a potent activator of enzymatic and non-oxidative ROS scavenging systems and induces stimulation of mitochondriogenesis in muscle [153]. A moderate level of oxidative stress, as occurs in non-exhaustive exercise, up-regulates PGC1α by promoting oxidative fiber formation at the expense of glycolytic fiber formation, increasing muscle mass and strength and resistance to muscle wasting, together with enhancing the early stages of adult muscle stem cell activation and proliferation [154]. In this scenario, irisin, which is a myokine induced by physical activity and which is involved in energy expenditure, insulin sensitivity and anti-inflammatory pathways, could play a key role. However, this myokine improves mitochondrial function and reduces ROS production. As shown in Figure 2, irisin seems to protect skeletal muscle against metabolic stresses, including oxidative stress, but the mechanism is almost completely unknown [155]. In a study carried out on a mouse myogenic cell line (C2C12), myoblasts in which irisin was overexpressed by transfection were observed to have a significant increase in cell viability and a decrease in apoptosis induced by increased glucose [156]. More closely related to mitochondrial alteration and possible ROS accumulation, irisin overexpression also appears to inhibit glucose-induced suppression of the increase in mitochondrial membrane potential [157].

In vitro experiments performed on H9c2 cardiomyocytes to mimic myocardial remodeling also showed that irisin treatment in the presence of H_2_O_2_ attenuated intracellular ROS levels and cardiomyocyte apoptosis in a dose-dependent manner. This occurs because miR-19b irisin-dependent expression can reactivate the AKT/mTOR signaling pathway blocked by H_2_O_2_ in H9c2 cells. Taken together, these data provide new insight into the mechanism by which irisin may have beneficial effects on myocardial remodeling [158].

When we try to interpret these apparently contradictory data, we need to reflect on what Nikolaos Perakakis and his collaborators wrote “When interpreting the results of these exercise-based studies, one must remember that a high degree of heterogeneity exists between study designs, which makes reliable and generalizable conclusions difficult. For example, some studies that used chronic-exercise protocols were unable to detect changes in circulating levels of irisin, but these findings should not be interpreted as a lack of effect of exercise on irisin secretion. Moreover, studies that did not show that PGC1α was upregulated by exercise might have not used the appropriate experimental model to investigate the relationship between irisin and exercise. Furthermore, most human studies had few participants, and their results were based on commercially available antibody tests that have been questioned for their sensitivity” [130].

Figure 2 summarizes the mechanism of action proposed for the selected myokines, specifically in correlation with oxidative stress. In particular, MGF, IGF-1, S100 and irisin are able to counteract oxidative stress, thus improving mitochondrial function and reducing ROS production; conversely, Myostatin increases oxidative stress that in turn increases the myostatin level. Hence, depending on the positive or negative modulation of a specific myokine level produced by muscle secretome, it is possible to observe an anti-aging effect not only in the skeletal muscle but also widespread throughout the body.

## 3. Concluding Remarks

In conclusion, even taking into account the multifactorial nature of the etiopathogenesis of sarcopenia (assuming that this state can be defined as pathological), there is now a general consensus that the imbalance of ROS in muscle cells, caused by defective control of mitochondrial homeostasis, reduced physical activity and/or an excess of caloric intake, is one of the main causes of the cellular aging process. ROS imbalance occurs in myofibers, causing metabolic events that lead to an imbalance in protein synthesis with the onset of muscle atrophy. However, ROS imbalance could in turn lead to the reduced regenerative capacity of stem cells responsible for maintaining skeletal muscle mass and to the depletion of the reserve pool of satellite cells. Outside muscle cells, extrinsic factors, including some myokines associated with the niche, and intrinsic cell-autonomous factors contribute to determining and/or counteracting age-related changes in muscle cells.

Based on data collected from many laboratories, we infer that, among the myokines discussed here, irisin could be one of those most involved in regulating the oxidative state, mitochondrial genesis and the repair of cellular structures damaged by contractile activity that occurs in the presence of oxidative stress.

Although the available data are certainly insufficient to clearly delineate the protein’s mechanism of action, they indicate that the availability of irisin (which does not act only in skeletal muscle) is directly proportional to its antioxidant capacity. The levels of this myokine are undoubtedly reduced in various conditions, both physiological, such as senescence, and pathological, such as insulin resistance and myocardial disruption. Its plasma concentration, however, can be easily recovered because physical activity can, at any age, increase its presence and availability.

Of course, irisin is not the only product of the muscle secretome able to drive, through both autocrine and paracrine and/or endocrine action, progression towards the senescent phenotype of the muscle, but it is interesting for at least three characteristics:

(1) Specific action against oxidative stress;

(2) Widespread action throughout the body;

(3) The possibility that its plasma level can be increased simply by increasing physical activity.

For this reason, a better understanding of the mechanisms of action of irisin could be the starting point to characterize this myokine as a fundamental factor in counteracting senescence-related decay, at least in muscle tissues.

## Figures and Tables

**Table 1:** Myokines linked to senescence-related changes.

Myokine	Principal Targets	Specific Membrane Partners	Intracellular Effect on Muscle	Regulation by Physical Exercise	Modulation by Muscle Aging	Possible Effects on Muscle Aging
Myostatin	Muscle (skeletal and cardiac), adipose tissue, brain	ActRII/B and TGFβRII receptors	Inhibition of protein synthesis and regenerative processes	inhibited	increased	inflammation and oxidative stress
NGF	Muscle (skeletal and cardiac), brain	TrkA and p75NTR receptors	Stimulation of regenerative processes	increased	increased/decreased	increase in the presence of type I fibers
IGF-1	Muscle (skeletal and cardiac), bone, adipose tissue	tyrosine kinase receptors (IGF-1 and IGF-2)	Stimulation of protein synthesis and regenerative processes, inhibition of catabolic pathways	increased	increased/decreased	alteration of IGF/IGFR system
S100	Muscle (skeletal and cardiac), brain	RAGE, G-protein-coupled receptors, *N*-glycans	Regulation of Ca^2+^-dependent mechanisms and regenerative processes	increased	decreased in myoblasts	limitation of regenerative processes
Irisin	Muscle, bone, adipose tissue, cardiovascular system	αV/β5 integrins (bone, adipose tissue)	Thermogenesis, glucose homeostasis, mitogenesis	increased	decreased	decreases stimulation of mitochondrial biogenesis

The table shows the myokines selected according to the following criteria: (1) the manifest ability of the myokine to act both from the inside of the cell and in an autocrine fashion; (2) the existence of a definite relation between the presence of the myokine with the modulation of the ROS balance of the fibers involved in regulatory processes (metabolic or regenerative) of muscle aging. More information on the listed myokines is described in specific paragraphs.
